# Sulfur/Organic Copolymers as Curing Agents for Rubber

**DOI:** 10.3390/polym10080870

**Published:** 2018-08-05

**Authors:** Jakub Wręczycki, Dariusz M. Bieliński, Rafał Anyszka

**Affiliations:** 1 Institute of Polymer and Dye Technology, Faculty of Chemistry, Lodz University of Technology, 90-924 Lodz, Poland; dariusz.bielinski@p.lodz.pl (D.M.B.); rafal.anyszka@p.lodz.pl or r.p.anyszka@utwente.nl (R.A.); 2 Department of Mechanics of Solids, Surfaces & Systems (MS3), Faculty of Engineering Technology, University of Twente, 7500 AE, Enschede, The Netherlands

**Keywords:** sulfur, sulfur polymers, inverse vulcanization, elastomer, rubber, curatives, crosslinking, crosslink density, freezing point depression

## Abstract

It is widely acknowledged that waste sulfur generated from the petroleum industry creates huge storage and ecological problems. Therefore, the various methods of utilization are becoming increasingly attractive research topics worldwide. The thermal ability of elemental sulfur to homolytic cleavage of S_8_ rings enables its free radical copolymerization with unsaturated organic species and the obtaining of chemically stable polymeric materials. Here we report a novel possibility to use sulfur/organic copolymers obtained via “inverse vulcanization” as curatives for rubber. For this purpose, several various sulfur/organic copolymers were synthesized and analyzed from the point of view of their performance as rubber crosslinking agents. Solvent extraction was used to purify sulfur/organic copolymers from unreacted (elemental) sulfur. Thermal properties of the prepared copolymers were characterized by thermogravimetric analysis and differential scanning calorimetry (TGA–DSC). Crosslink density and structure of cured elastomers was studied by equilibrium swelling, thiol-amine analysis and freezing point depression. Mechanical properties of the vulcanizates were determined under static and dynamic conditions (DMA—dynamic mechanical analysis). It is proved that the utilization of sulfur/organic copolymers as curatives enables an effective crosslinking process of rubbers. Taking into account the results of a crosslink density analysis and mechanical properties of the vulcanizates cured with purified copolymers, it is evident that relatively long copolymer macromolecules are also involved in the formation of chemical bonds between unsaturated rubber macromolecules.

## 1. Introduction

Elemental sulfur occurs in nature in a pure (native) form or in a form of various sulfur-containing minerals (sulfides or sulfates), but it is also a by-product from petroleum industry, mainly from hydro-desulfurization process. Hence, enormous amounts of sulfur are generated annually as waste. The necessity for sulfur removal in petroleum refining processes is strictly related to rigorous requirements for sulfur content in diesel fuels (even up to 5 ppm) [1,2]. A very promising and environmentally beneficial approach to utilize waste sulfur is associated with its thermal behavior: the ability to transformation into chemically stable polymeric materials [2].

The most thermodynamically stable form of sulfur under ambient conditions is orthorhombic sulfur (S_α_), present in the form of eight-membered rings (S_8_ or cycloocta-S). Heating of orthorhombic S_8_ leads to its conversion into monoclinic S_8_ (S_β_) at 95.6 °C under atmospheric pressure. Further heating of S_8_ rings results in melting of solid state sulfur (mainly S_β_ and residual S_α_ crystals), leading to obtaining a yellow liquid (S_λ_) at melting point temperature of around 119 °C. S_λ_ consists predominantly of S_8_ rings. At temperatures above 159 °C S_8_ monomer rings present in a deep-red liquid (above this temperature the melted sulfur is described as S_μ_) exhibit the ability to undergo spontaneous ring-opening polymerization (ROP), resulting in the formation of linear sulfur diradicals which polymerize into a polymeric form of sulfur (S_w_). If the polymerized mixture is quickly quenched to ambient temperature a product which can be described as “plastic sulfur” is obtained. However, the sulfur-polymer material obtained from the reaction consists predominantly of unreacted sulfur in form of S_8_ and contains merely a few percent of a polymeric phase (a polymeric form of sulfur, also called insoluble sulfur (IS)). The ratio of the polymeric phase to eight-membered sulfur rings is strictly related to conditions of polymerization process, in particular to reaction temperature, time and conditions of cooling (cooling rate, heat transfer from the bulk of polymeric phase, etc.). The polymeric form of sulfur as opposed to elemental sulfur is insoluble in carbon disulfide (CS_2_) and toluene, therefore it is possible to separate insoluble sulfur by extracting S_8_ with CS_2_ or toluene. However, in the course of time a sulfur-polymer material becomes brittle and exhibits poor mechanical properties due to the depolymerization process of the polymeric phase leading to the re-formation of S_8_ rings and consequent recrystallization of S_8_ to orthorhombic form (S_α_) [2,3,4,5,6].

The insoluble sulfur may be stabilized during polymerization process when sulfur diradicals are thermally generated. One of the most well-known approaches, which has been studied since the 1970s by B. R. Currell et al. [5], L. Blight et al. [7], B. K. Bordoloi and E. M. Pearce [8], is to conduct a copolymerization of elemental sulfur with unsaturated organic compounds, i.e., dicyclopentadiene, styrene or limonene via a bulk copolymerization process. This leads to the synthesis of sulfur/organic copolymers. Recently this process was accurately described and nicknamed as “inverse vulcanization” by W. Chung and J. Pyun et al. [2] because of its contrary nature to classic sulfur vulcanization of rubber. This has drawn new attention to sulfur-based copolymers for various advanced materials applications [9,10,11,12].

Vulcanization is a process leading to the formation of a three-dimensional elastomer network via chemical crosslinking reactions between linear macromolecules of natural or synthetic rubbers and curing agents (typically sulfur or peroxide systems). This process is necessary to decrease the plasticity of rubber compositions and, most importantly, simultaneously increasing their elasticity, which allows practical applications of rubber goods. Vulcanization of rubber with elemental sulfur, which is the most common vulcanizing agent used for crosslinking of unsaturated rubbers (i.e., NR—natural rubber, SBR—styrene-butadiene rubber or NBR—nitrile-butadiene rubber), was discovered by C. Goodyear and by T. Hancock over 170 years ago and found widespread industrial implementation [3,13]. The efficient vulcanization process requires incorporation of a relatively high amount of sulfur. However, rubber compositions containing high sulfur amount may exhibit an undesirable sulfur migration effect from the bulk to their surface. This might occur during storage or after vulcanization process if a considerable amount of poorly dispersed sulfur remains unreacted due to its poor solubility in a nonpolar rubber matrix [4,14]. This phenomenon is commonly known as “sulfur blooming” and has been substantially discussed in the subject literature [4,14,15].

Sulfur exhibits a tendency to bloom onto rubber surface if its concentration exceeds maximum solubility in the rubber at storage temperature [4,14,16]. A white bloom on rubber mix surface is associated with sulfur crystallization and causes loss of tackiness between two layers of rubber compounds. It is likely to adversely affect, for example, a tire forming process in which rubber compound tackiness plays a very important role [4,14,17].

There are two types of sulfur commonly used in the rubber industry: elemental (in-rubber soluble) sulfur and polymeric (in-rubber insoluble) sulfur. The most commercially known product based on polymeric sulfur is Crystex™. However, there is no available information about its chemical composition explaining the name. This is how a new potential area to utilize sulfur/organic copolymers emerges. Despite there being few reports on sulfur/organic copolymers application for elastomeric composites in scientific papers, some examples exist in the patent literature [14,18,19,20,21].

H. Colvin and Ch. Bull. Jr. investigated rubber vulcanizates crosslinked with sulfur/olefin reaction products obtained via a suspension polymerization process. The products are mixtures of polysulfide macromolecules and free sulfur which exists in the form of monoclinic sulfur and amorphous supercooled liquid sulfur. The authors claim that the polysulfide macromolecules exhibit stabilized properties on these forms of free sulfur and mechanical properties of the vulcanizates depend primarily on the free sulfur content in the sulfur/olefin adducts [4,14]. W. Chung and J. Pyun et al. also mentioned the possibilities for utilizing these materials in tire manufacturing processes [2].

The aim of this work is to determinate the chemical composition and macromolecular structure of sulfur-organic copolymers, and their optimization from the point of view of their performance as rubber crosslinking agents. The crosslink density, structure and the degree of modification of rubber macromolecules were studied and compared to mechanical properties of the vulcanizates under static and dynamic conditions.

## 2. Materials and Methods

### 2.1. Materials

Elemental (rhombic) sulfur in the form of powder was received from Siarkopol Tarnobrzeg (Tarnobrzeg, Poland). Organic comonomers used were provided by: dicyclopentadiene (DCPD)—(≥95%, Sigma-Aldrich, Saint Louis, MO, USA), furfural (FUR)—(99% Sigma-Aldrich), turpentine (TRP)—(technical grade, POCH, Gliwice, Poland), styrene (ST)—(99%, Acros Organics, Morris Plains, NJ, USA), liquid rubber Thiokol LP23 (LP23)—(Toray Industries, Tokyo, Japan) and limonene (LIM)—synthesized by the Institute of Organic Chemistry (Lodz University of Technology, Lodz, Poland). Cold emulsion styrene-butadiene rubber (SBR) KER 1500 with 23.5% of bound styrene was obtained from Synthos (Oświęcim, Poland) and chosen as an example of the most utilized synthetic rubber. Zinc oxide (ZnO), stearic acid and vulcanization accelerator—*N*-cyclohexylbenzothiazole-2-sulfenamide (CBS)—was purchased from Lanxess (Cologne, Germany). Toluene for crosslink density analysis as well as the determination of insoluble (polymeric) sulfur content in sulfur/organic copolymers was obtained from POCH (Gliwice, Poland). The reagents for thiol-amine analysis: piperidine (99%), 2-propanethiol (≥98%), 1-dodecanethiol (≥98%) were provided by Sigma-Aldrich, Diethyl ether was purchased from Sigma-Aldrich, Cyclohexane (standard for chromatography) was received from Z. D. Chemipan (Warsaw, Poland).

### 2.2. Preparation of Sulfur/Organic Copolymers

Sulfur-organic copolymers were synthesized via bulk copolymerization process (inverse vulcanization). The synthesis was conducted in a glass reaction vessel of 250 mL volume, covered with a four-necked glass lid. The glass lid was equipped with a mechanical stirrer, a thermocouple, a condenser and a dropping funnel. The reactor was immersed in a silicone oil heating bath and the sulfur was heated up to appropriate temperature. Each time the organic comonomers were dripped into the heated (liquid) sulfur within 30 min. The inverse vulcanization process was carried out under the air atmosphere and atmospheric pressure. The obtained products were poured out into silicone molds at room temperature.

### 2.3. Determination of Insoluble (Polymeric) Form of Sulfur Content in Sulfur/Organic Copolymers

The determination of insoluble (polymeric) sulfur content in sulfur/organic copolymers was carried out according to the ASTM D 4578—89 (2002) standard test method [22]. In this test, toluene was used as a solvent for soluble (elemental) sulfur during extraction.

### 2.4. Thermal Properties of Sulfur/Organic Copolymers

Thermogravimetric analysis (TGA) was conducted using the Netzsch TG 209 analyzer (Netzsch, Selb, Germany) and Differential Scanning Calorimetry (DSC) was carried out using the Netzsch DSC 204 device (Netzsch, Selb, Germany). Both analyzers were connected with the Netzsch TASC 414/3A heat controller (Netzsch, Selb, Germany). The TGA tests were performed on small powder samples (9–10 mg) using aluminum (Al) crucibles under nitrogen atmosphere. The tested temperature range was 40–450 °C applying a heating mode and rate of 10 °C/min. DSC analyses were performed under nitrogen atmosphere in temperature range from 40 to 200 °C. The samples were tested in aluminum (Al) crucibles with heating rate of 7.5 °C/min (in the temperature range from 40 to 80 °C and from 140 to 200 °C) and 2.5 °C/min (in the temperature range from 80 to 140 °C). The decrease of heating rate in the temperature range from 80 to 140 °C was applied to provide better peaks resolution that occurred in this range.

### 2.5. Preparation of Rubber Mixes

The rubber mixes were prepared in the internal laboratory micromixer Brabender Plasti-Corder (Brabender, Duisburg, Germany). Firstly, SBR was placed in a mixing chamber (75 mL) and plasticized for 2 min (30 rpm). After that, stearic acid and zinc oxide (ZnO) were added during another 2 min (30 rpm) and homogenized for the next 1 min (40 rpm). Afterwards, within 2 min accelerator and elemental (rhombic) sulfur, sulfur polymer or sulfur/organic copolymers were incorporated (30 rpm). After 3 min of mixing (40 rpm) the rubber mix was pulled out from the mixing chamber. The total mixing time was 10 min. After a few hours of conditioning, the rubber mixes were rolled into sheets with the David Bridge (UK) two-roll open mixing mill operating with friction of 1.1 during 2–3 min.

The compositions of particular rubber mixes are given in Table 1. The difference between content of curatives in samples marked as S/D and S/D/(e.g., ST) in comparison to a reference sample (S) crosslinked with elemental sulfur and sample SP is a result of the fact that sulfur/organic copolymers already containing sulfur and organic fractions originated from particular comonomers. Therefore, a little excess is essential to keep up the same sulfur level of 1.52 phr (parts per hundred rubber) in every rubber mix studied.

The proposed amounts were also maintained for the copolymers after purification in order to evaluate how it influences the rubber samples properties. Nevertheless, the sulfur/organic comonomer ratio changed after the purification.

### 2.6. Kinetics of Vulcanization and Vulcanization Process

Studies on vulcanization kinetics of the SBR rubber-based mixes were conducted by means of the MDR 2000 (Alpha Technologies, Akron, OH, USA) rheometer. The bottom rotor was working with the standard frequency of 1.667 Hz (100 cpm). Optimum vulcanization time (τ_0,9_) according to ISO 6502 testing standard at the temperature of 160 °C as well as an increase of the torque were determined. The SBR mixes were vulcanized in steel molds placed in an electrically heated laboratory press at 160 °C and under pressure of 10 MPa, for a period of time calculated from the parameters of vulcanization kinetics.

### 2.7. Crosslink Density Analysis by Equilibrium Swelling (Flory–Rehner Equation)

The total crosslink density of the rubber samples (*ν*) was determined by equilibrium swelling in toluene and calculated on the basis of the Flory–Rehner Equation (1) [23]. Five samples (0.03–0.05 g) were cut out from each analyzed vulcanizate. After weighing, the samples were placed into closed weighing bottles with toluene. The weighing bottles with samples were left for 48 h at room temperature (25 ± 1 °C), to reach swelling equilibrium. After that, the swollen samples were immersed one by one for a few seconds in diethyl ether, which was followed by drying of their surfaces carefully with a blotting paper. The swollen samples were weighed. After drying the samples to constant mass in a vacuum oven (at 60 °C for 72 h), they were weighed again. Flory–Huggins coefficient (*χ*) for sulfur crosslinked SBR–toluene pair used in the equation was 0.37. Determined SBR density was 0.94 g/cm^−3^.

(1) $$v=-\frac{ln\left(1-V_{r}\right)+V_{r}+\chi V_{r}^{2}}{V_{0}\;\left({V_{r}}^{\frac{1}{3}}-\frac{2V_{r}}{f}\right)}$$

where: *ν*—crosslink density per unit volume (mol/cm^3^); *V_r_*—volume fraction of rubber in a swollen sample [-]; *V_0_*—solvent molar volume (for toluene: *V_0_* = 106.9 cm^3^/mol); f—functionality of crosslinks (*f* = 4, assuming the formation of tetra-functional crosslinks); *χ*—Flory–Huggins rubber-solvent interaction parameter (0.37 for sulfur crosslinked SBR–toluene pair).

### 2.8. Crosslink Density and Structure Analysis by Crioscopic Method (Freezing Point Depression Experiments)

The freezing point depression experiments were performed using the Netzsch DSC 204 analyzer (Netzsch, Selb, Germany) connected with the Netzsch TASC 414/3A heat controller (Netzsch, Selb, Germany) and the Netzsch cooling controller CC200 (Netzsch, Selb, Germany). The samples of the vulcanizates used in these experiments were the same as used for the crosslink density analysis by equilibrium swelling in toluene. The prior equilibrium swelling procedure resulted in the removal of non-bound polymer chains and small-molecular contaminations, leaving only interconnected by crosslinks polymer chains. The dried samples were swollen to equilibrium mass in cyclohexane for at least 48 h at room temperature (25 ± 1 °C). Afterwards, the small piece of swollen vulcanizate (approx. 10 mg) was transferred into aluminum (Al) crucible suitable for a DSC analysis and the pure cyclohexane was also dripped in an excess to the crucible to ensure that the sample was totally submerged in the solvent. The crucible was sealed with the lid and pressed. The measurements were carried out with cooling rate of 5 °C/min in the temperature range from 25 to −45 °C under nitrogen atmosphere. By comparison of the freezing temperature of solvent confined (trapped) in the rubber network (swollen to equilibrium) and a free solvent surrounding the rubber sample (solvent in DSC crucible) it is possible to calculate a freezing point temperature depression (∆*T*), which indicates the degree of crosslinking. An in-depth explanation of this method and the basic dependences between ∆T and crosslink density of rubber are described and discussed in Section 3.4.2.

### 2.9. Crosslink Structure Analysis by Thiol-Amine Analysis

Thiol-amine analysis was carried out using two sets of thiol-amine reagents: “soft” and “hard”, according to the procedure developed and described by B. Saville and A. Watson [24]. “Soft” thiol-amine reagent cleaves strictly polysulfidic crosslinks (C–S_x_–C, x > 2), therefore remaining after this treatment are: disulfidic (C–S_2_–C), monosulfidic (C–S–C) and carbon-carbon (C–C) crosslinks. “Hard” thiol-amine reagent cleaves polysulfidic (C–S_x_–C, x > 2) and disulfidic crosslinks (C–S_2_–C). Therefore, crosslinks remaining after this treatment are monosulfidic (C–S–C) and carbon-carbon (C–C) crosslinks. The “soft” thiol-amine reagent was prepared as a mixture of 2-propanethiol (0.4 M) and piperidine (0.4 M) in toluene. The analysis based on “soft” reagent was conducted for 2 h on pre-swollen vulcanizates (12 h in toluene) in a closed glass vial under nitrogen atmosphere. The “hard” thiol-amine reagent was prepared as a mixture of 1-dodecanethiol (1.0 M) and piperidine [25]. The analysis based on the “hard” reagent was conducted for 24 h on vulcanizates in a closed glass vial under nitrogen atmosphere. After both analysis the samples were removed instantly from the probes and washed at least a few times in order to get rid of reagents residues using toluene (15 min). Afterwards, the samples were swollen to equilibrium mass in toluene according to the crosslink density analysis procedure by equilibrium swelling. The remaining crosslink density was calculated from the Flory–Rehner equation.

### 2.10. Mechanical Properties of Vulcanizates under Static Conditions

Mechanical properties of the vulcanizates under static conditions (tensile strength (*TS*), elongation at break (*E*_b_) and modulus at 100% of elongation (SE100)) were investigated with the use of the universal static testing machine Zwick Roell type 1435 equipped with mechanical extensometers (Zwick Roell, Ulm, Germany). The measurements were carried out on dumbbells type 3 (ISO 37) specimens (four for each sample) at temperature of approx. 23 ± 1 °C with the constant crosshead speed of 500 mm/min.

### 2.11. Dynamic Properties of Vulcanizates

Dynamic mechanical analysis (DMA) was carried out with the use of a DMA/SDTA861e analyzer (Mettler Toledo, Greifensee, Switzerland), operating in the tension mode. Measurements of DMA parameters (storage modulus (*M*’), loss modulus (*M*”) and loss factor (tanδ)) were conducted in temperature range from −120 to 80 °C with heating rate of 3 °C/min, strain amplitude of 4 μm and frequency of 1 Hz. The glass transition temperature of the analyzed samples was determined from the maximum value for their tanδ = f(*T*) functions.

## 3. Results and Discussion

### 3.1. Synthesis of Sulfur/Organic Copolymers

Destined as curing agents for rubber, sulfur polymer (SP) and eight various sulfur/organic copolymers were synthesized. The synthesis procedure was described in Section 2.2. Conditions of (*co*)polymerization processes as well as the composition of prepared curatives are provided in Table 2.

In most cases similar conditions of (*co*)polymerization processes were applied, except the samples S/D_160/140/3_ and S/D_160/140/6_. Conventionally, elemental sulfur was firstly heated up to temperature of approximately 140 °C and after the temperature of liquid sulfur was stable, dicyclopentadiene (DCPD) or its mixtures with another comonomer (e.g., styrene) were dripped into liquid sulfur during 30 min. (*Co*)polymerization processes were run for 3 h. An alternative approach was developed in the case of the sample S/D_160/140/3_ and S/D_160/140/6_. At first, elemental sulfur was heated up to 160 °C to ensure that majority of the eight-membered rings were open, and then polymerized for 1 h. Afterwards the temperature was decreased to 140 °C and dicyclopentadiene (DCPD) was dripped into the liquid sulfur and copolymerization process was continued for 3 h (S/D_160/140/3_) or 6 h (S/D_160/140/6_). In case of sulfur polymer (SP), elemental sulfur was heated up to 160 °C and then polymerized for 3 h.

All the obtained products were rather low or moderately viscous liquids, which were able to be poured out to silicone molds. Depending on the organic comonomers that were used in the synthesis process, the liquid products and the solids had different colors. In the case of copolymers based on sulfur and DCPD (S/D_140_, S/D_160/140/3_ and S/D_160/140/6_) the liquids were dark amber-like and brown in color. After cooling down the obtained solids had dirty yellow shades. Similar color changes were observed in the case of copolymers S/D/ST and S/D/LP23. Copolymers based on turpentine (S/D/TRP) as well as limonene (S/D/LIM) were deep-brown in color in the form of liquids. After cooling down the solids exhibited a slightly darker shade of yellow (orange/yellow). Interesting colors were noticed in the case of copolymer based on furfural (S/D/FUR). The liquid was dark brown/black, which is related to the monomeric furfural color (black) and afterwards the solids became khaki-like. Liquid sulfur polymer (SP) was red-amber in color. After pouring out to the mold and cooling down the red-amber color was retained for a few minutes. However, afterwards small yellow spots started to appear and after a few hours the whole surface became yellow. In this case, clearly visible crystals were noticed on the surface. Once the prepared (*co*)polymers became solid-state, they were crushed up with the use of mortar and pestle in order to prepare powders ready to use as curing agents for rubber.

### 3.2. Determination of Mass Ratio of Elemental (Soluble) Sulfur and Polymeric (Insoluble) Sulfur in Sulfur/Organic Copolymers

**Figure 1:**
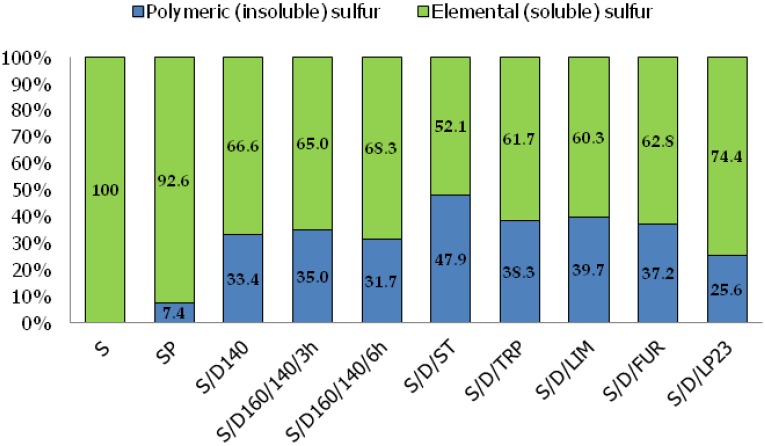
The mass ratio of elemental (soluble) sulfur and polymeric (insoluble) form of sulfur in sulfur/organic copolymers.

The investigation of a polymeric (insoluble) form of sulfur in sulfur/organic copolymers is crucial to draw conclusions about their behavior and impact during the rubber curing process. As it has been described, the mass ratio of elemental (soluble) and polymeric (insoluble) form of sulfur was investigated according to ASTM D4578 standard in which toluene was used as a solvent for soluble sulfur. The results are reported in Figure 1.

As was expected, sulfur polymer (SP) contains merely 7.4 wt % of sulfur in the form of macromolecules (polymeric phase). This can be explained by the fact that polymeric phase is unstable under ambient conditions and quickly recombines back to the monomeric form of crystalline orthorhombic sulfur (S_α_). What is more, the analysis was performed at least several days after the synthesis process, which might have depressed the initial content of the polymeric phase in case of SP. This result suggests that the sulfur polymer may behave mostly like elemental sulfur rather than polymer during the curing process. However, in copolymer compositions the insoluble sulfur content fluctuates in the range from 25.6 wt % (S/D/LP23) to 47.9 wt % (S/D/ST) with the average value of approximately 31 wt %. On the one hand, such high content of the polymeric phase is a result of the stabilization process, due to sulfur copolymerization with unsaturated organic species, which significantly raises the content of macromolecules in comparison to SP. On the other hand, the polymeric phase content is in general quite low, probably due to the equilibrium restrictions occurring during spontaneously (thermally) initiated bulk copolymerization.

D. L. Hammick, W. R. Cousins and E. J. Langford [26,27] as well as J. Schenk [27,28] attempted to determine the polymeric phase content in a quickly quenched sulfur homopolymerization mixture. They measured the weight fraction of insoluble sulfur in carbon disulfide. The highest weight fraction of polymeric phase (insoluble sulfur) in sulfur homopolymer obtained by them was approximately 40 wt %. This is due to the fact that sulfur exhibits relatively poor heat conductivity, hence it is not possible to quickly and homogenously drop the temperature of a reaction mixture by instant freezing in order to preserve all polymeric form of sulfur. Additionally, in the case of copolymerization of elemental sulfur with vinyl monomers (e.g., styrene), the limited content of polymeric phase may be ascribed to the fact that sulfur macroradicals recombine much faster than addition of vinyl monomer to the polymer chain takes place [29]. For this reason, obtaining more than ~50 wt % of the polymeric phase, even in the case of sulfur/organic copolymers, seems to be really challenging when using bulk copolymerization.

B. R. Currell et al. [5] investigated the content of the polymeric phase in sulfur/copolymers based on various olefins (i.e., myrcene, alloocimene, limonene) and selected liquid Thiokol rubbers as comonomers synthesized via bulk copolymerization. The amount of each of organic comonomer was 25 wt %. The temperature of the copolymerization process was 140 °C and the time of copolymerization was 3 h. The content of polymeric phase in these materials was oscillating in the range from 26.0 wt % to 53.7 wt % (values measured after 18 months of storage at ambient temperature; there are no values provided immediately after synthesis process). The results obtained in our work are very similar to the results obtained by B. R. Currell et al.

H. Colvin and Ch. Bull. Jr. synthesized sulfur/organic copolymers based on dicyclopentadiene, styrene and divinylbenzene as comonomers via a suspension copolymerization process [4]. The content of polymeric phase in the products obtained by them (in the form of beads) was fluctuating in the range from 34.2 wt % to 82.9 wt % depending on used the comonomer, suspending agent and its content as well as copolymerization conditions. As can be seen in some cases this method allows to obtain products with higher polymeric phase content than products obtained via a bulk copolymerization process.

In this work, for each synthesized copolymer the polymeric phase contents were investigated in order to establish how its presence influences the properties of the rubber mixes. Moreover, the polymeric phases that remained after purification of the copolymers were also utilized in this work as curatives for rubber. The aim of this comparison is to obtain unambiguous information about the participation of sulfur/organic macromolecules in the rubber curing process and to investigate their influence on crosslink morphology and rubber properties.

### 3.3. Thermal Properties of Sulfur/Organic Copolymers

#### 3.3.1. Differential Scanning Calorimetry (DSC)

**Figure 2:**
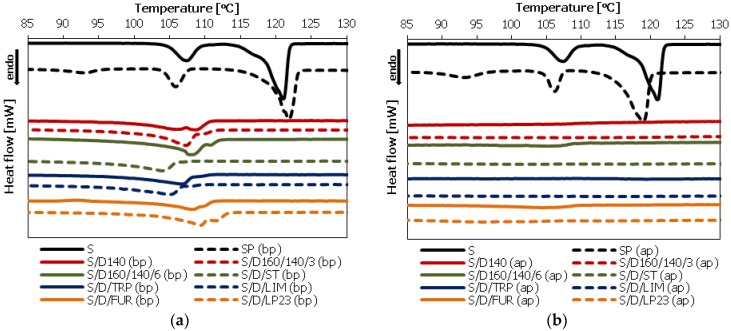
Differential scanning calorimetry (DSC) thermograms of synthesized sulfur/organic copolymers and their comparison with elemental sulfur: (**a**) before the purification process (extraction of soluble sulfur with toluene) (bp); (**b**) after the purification process (ap).

Differential Scanning Calorimetry (DSC) analysis was conducted to investigate the phase structure of the copolymers as well as to analyze the purification process results. The DSC measurements were carried out for the sulfur/organic copolymers before purification (before extraction of unreacted sulfur with toluene) as well as after purification. The comparison of the DSC thermograms is presented below, in Figure 2.

Elemental (crystalline) sulfur (S) exhibits two endothermal peaks during heating. The first of them occurs at temperature of approximately 107 °C and corresponds to the changes in the crystalline structure of sulfur from orthorhombic (S**_α_**) to monoclinic (S**_β_**). The second one, which occurs at temperature of approximately 120 °C, is related to the melting of S_β_ crystals and residual S_α_ crystals into yellow liquid (S_λ_). A similar situation is observed in the case of sulfur polymer (SP) before (bp) as well as after the purification process (ap), because the content of macromolecules (polymeric phase) in sulfur polymer is very low, due to their instability under ambient conditions, and it contains crystalline sulfur even after the purification procedure. In this case, the peaks originated from the crystalline phase (unreacted sulfur) are also clearly visible.

In the case of the copolymers before purification (bp), their thermograms (Figure 2a) also reveal the presence of unreacted (non-polymerized) sulfur, which is visible as endothermic (melting) peaks in the temperature range of 100–120 °C. However, the extraction of unreacted (soluble) sulfur with toluene results in pure (amorphous) sulfur/organic copolymers, as confirmed by DSC analysis of the copolymers after the purification process (Figure 2b). In every case the endothermic peaks originated from crystalline sulfur disappeared completely. This confirms high efficiency of the purification process.

#### 3.3.2. Thermogravimetric Analysis (TGA)

**Figure 3:**
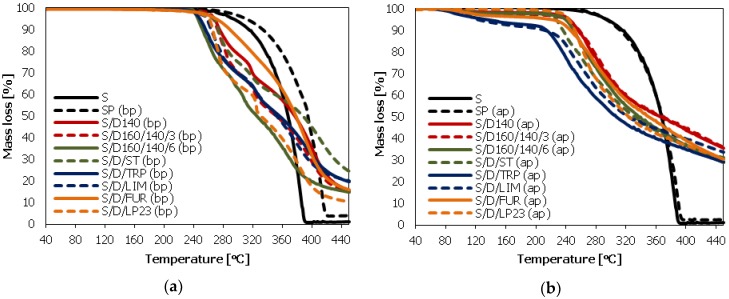
Thermogravimetric analysis (TGA) curves of the synthesized sulfur/organic copolymers and their comparison with elemental sulfur: (**a**) before the purification process (extraction of soluble sulfur with toluene) (bp); (**b**) after the purification process (ap).

Thermal stability of elemental sulfur and the prepared sulfur/organic copolymers was investigated by Thermogravimetric Analysis (TGA). The comparison of the TGA thermograms for the copolymers before and after the purification process are given in Figure 3a,b respectively.

Figure 3a shows the onset temperature of thermal decomposition of non-purified sulfur/organic copolymers, which initiates at around 220 °C. Sulfur and sulfur polymer outperform onset decomposition temperature in comparison to sulfur/organic copolymers. However, in their case at temperature of approximately 390 °C (S) and 420 °C (SP) (bp) the samples decompose completely. Non-purified sulfur/organic copolymers exhibit a rather multi-step decomposition except the S/D/FUR copolymer. This is due to the fact that the unreacted sulfur present in the copolymers influences significantly the kinetics of their thermal decomposition. The mutual dispersion and distribution of crystalline and polymer phases in the copolymers are unknown on the micromorphological level. Therefore, the TGA curves exhibit non-stable kinetics during thermal decomposition. Additionally, the chemical structures of the polymer phases consisting of sulfur and organic comonomers are not well defined. Their molecular mass and its distribution, sulfur/organic comonomer ratio and structure may vary considerably, additionally influencing the kinetics of decomposition.

In the case of the purified copolymers, the onset of mass loss starts at much lower temperature (Figure 3b). This can be explained by the evaporating of residual toluene which was used for the extraction of elemental sulfur. According to the ASTM D 4578—89 (2002) standard [22] the samples were dried at 70 °C to remove the residual toluene. Comparing to the boiling point of toluene (110–111 °C), the drying temperature proposed in the standard seems to be too low. In future work we will propose a modification of this procedure in order to assure efficient removal of the residual toluene. The thermal decomposition kinetics of purified sulfur/organic copolymers are much more stable, which is clearly visible on the curves. Moreover, decomposition kinetics of the copolymers are similar, regardless of the synthesis procedure or the type of comonomer. Most probably the branched/crosslinked structure of sulfur/DCPD main component of the copolymer plays an essential role in thermal decomposition of the composites. Even the amount of residue at 440 °C is almost the same for all the purified copolymers, indicating high similarity of thermal properties of polymeric phases of all the composites. In addition, this also confirms that the purification process was performed successfully.

According to the work of J.C. Bear and co-workers, who investigated a composition and a microstructure of sulfur/1,3-diisopropenylbenzene copolymers after the annealing process (*T* >750 °C) under nitrogen atmosphere, it can be concluded that the residual material consists of porous carbon doped with the remaining sulfur atoms. The presence of sulfur that remains after extremely high temperature exposition is ascribed to the fact that long sulfur/organic macromolecules presumably undergo fragmentation due to the reversibility of widely occurred S–S bonds, particularly at elevated temperatures, and form shorter linkages that are more thermally stable. In that, in our case the content of residual materials at 440 °C is much higher in comparison to elemental sulfur (S) and sulfur polymer (SP) [30]. Furthermore, the occurrence of other bonds (e.g., C–S) present in chemical structure of sulfur/organic copolymers may also have an impact on their thermal stability.

### 3.4. Study of Crosslink Density and Network Structure of Rubber

#### 3.4.1. Equilibrium Swelling (Flory–Rehner Equation) and Thiol-Amine Analysis

**Figure 4:**
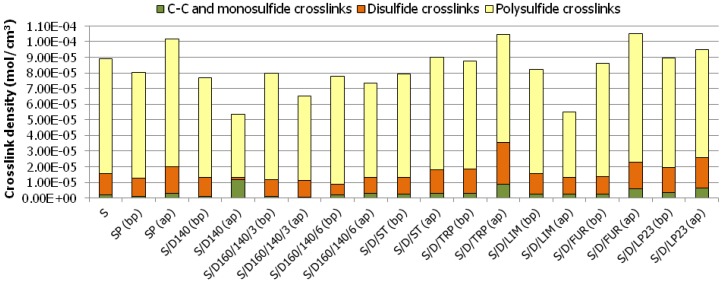
Crosslink density and crosslink structures of rubber vulcanizates cured with various curatives. Symbols in brackets refer respectively to particular sulfur/organic copolymers before purification (bp) and after purification (ap).

Curing of SBR rubber with various sulfur/organic copolymers before and after purification process results in the successful formation of vulcanizates with considerably diversified crosslink density. The results show clearly that curatives based on sulfur/organic copolymers are taking part in curing processes and are involved in the formation of chemical bonds between rubber macromolecules. The crosslink density calculated on the basis of Flory–Rehner equation as well as the participation of various crosslinks determined by thiol-amine analysis are presented in Figure 4.

**Figure 5:**
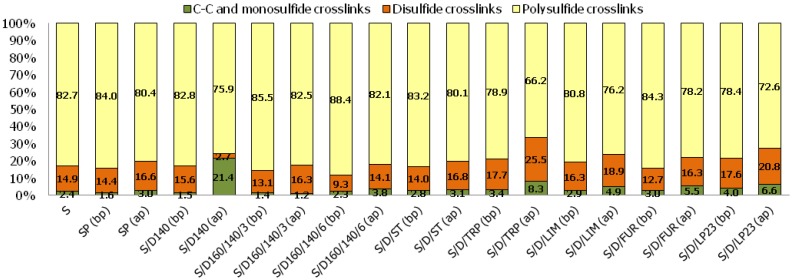
Crosslink structure of the rubber vulcanizates cured with sulfur/organic copolymers.

Figure 5 gives information on crosslink structure of the vulcanizates studied.

It is evident that in some cases the curing with sulfur/organic copolymers results in vulcanizates even with higher crosslink density than the reference sample cured with elemental (orthorhombic) sulfur, for example, in the case of samples cured with copolymers after the purification process, based on DCPD and turpentine (S/D/TRP (ap)), DCPD and styrene (S/D/ST (ap)), or DCPD and furfural (S/D/FUR (ap)) or DCPD and Thiokol LP23 (S/D/LP23 (ap)). Possibly, this is due to the fact that the curing process with purified sulfur/organic copolymers may occur without intramolecular modifications of the rubber chains, i.e., cyclic sulfidic structures, which is a common phenomenon for curatives based on elemental sulfur [31,32]. The size of the copolymers molecules is much higher than small eight-atom rings of crystalline sulfur. Therefore, it is more likely that they would connect two rubber macromolecules than modify the same macromolecule due to their own steric hindrance. Hence, higher amounts of curatives are able to take part effectively in the curing process and form chemical interconnections between rubber macromolecules.

Comparing the participation of various types of crosslinks in prepared vulcanizates, Figure 4 and 5 show that all cured samples contain predominantly polysulfidic crosslinks (no fewer than 66.2% in the case of sample S/D/TRP (ap)), moderate content of disulfidic crosslinks and moderately low content of monosulfidic and C–C crosslinks. In general, in most of the cured samples, the ratio between various crosslinks being formed is preserved constant and corresponds to the reference sample (S) cured with elemental sulfur. The exception is sample S/D140 (ap) which contains significantly higher monosulfidic and C–C crosslinks (21.4%) than other samples (on average 4.4%), which may influence mechanical properties of the vulcanizate.

What is more, as indicated in Figure 5, the samples cured with purified sulfur/organic copolymers contain less polysulfidic crosslink and simultaneously more disulfidic, monosulfidic and C–C crosslinks. This phenomenon may be an effect of a generally lower content of sulfur in purified sulfur/organic copolymers, since part of them in the form of low molecular sulfur (unreacted) was removed (dissolved in toluene) during extraction (purification procedure). Such crosslinks structure has a significant impact on the mechanical properties of the vulcanizates.

#### 3.4.2. Freezing Point Depression Experiments (Crioscopic Method)

Studies on a polymer network of elastomeric materials (crosslink structure) may be conducted by following the differences in the behavior of a solvent confined (trapped) in the rubber network (swollen to equilibrium) and a free solvent surrounding the rubber sample. By comparing the freezing temperature of both solvents it is possible to calculate a freezing point temperature depression (∆*T*). To investigate these changes differential scanning calorimetry (DSC) is used [33,34].

In general, ∆T is associated with the dimensions of free volumes (otherwise mesh size) of rubber network, between occurring crosslinks that in turn result from crosslink density. If the crosslink density of rubber is high, particular crosslinks in an ideal case are distributed near each other, hence the dimensions of free volume are lower. The solvent trapped in tighter polymer network is hardly able to crystallize smoothly and ought to exhibit lesser tendency to crystallize. The other way around, if the crosslink density of rubber is lower the crosslinks are apart and the mesh sizes are larger. Hence, the solvent ought to have more space to crystallize [33,34]. However, according to the literature data, depression (decrease) of the freezing temperature of the solvent trapped in a polymer network may have various origins and a few theories have been proposed over the years [35,36,37,38].

W. Kuhn and co-workers [35,36] claimed that the depression of the freezing temperature of the solvent trapped in the polymer network is associated with mechanical restrictions of the network (namely the mesh width) that enforce the size of forming crystals. Free solvent surrounding the sample does not show such restrictions.

B. B. Boonstra and co-workers [37] ascribed the decrease of freezing temperature of the solvent confined in the polymer network to the increased mobility of solvent molecules solvated around the polymer chains. Disordering the crystal lattice surrounding the polymer network results from the motions of the chains (macromolecules) that are still mobile when temperature goes down, until the glass transition temperature.

The theory of C. L. Jackson and G. B. McKenna [38] explains that the lowering of freezing temperature of the solvent trapped in the polymer network is associated both with mechanical restrictions of the polymer network (mesh dimensions) that inhibit the nucleation process of crystals and allow the formation of small crystals as well as with lower thermodynamic potential of the trapped solvent molecules.

Summarizing these theories, one can see that many factors may influence the behavior of the solvent trapped in the polymer (rubber) network due to its complexity. Therefore, the crioscopic method seems to be valuable just in estimating crosslink density of rubbers (qualitative analysis) and requires comparison with results from other methods (comparative analysis). A clear-cut reason of depression of the freezing temperature still remains questionable and unambiguous.

**Figure 6:**
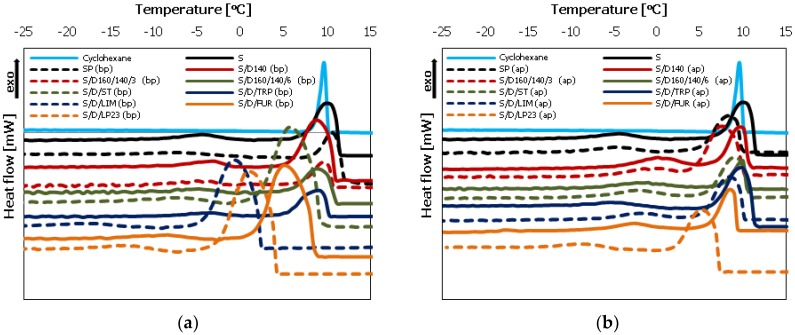
DSC thermograms of the freezing point depression for free cyclohexane and cyclohexane trapped in the swollen rubber network: (**a**) vulcanizates cured with the copolymers before purification process (extraction of soluble sulfur with toluene) (bp); (**b**) vulcanizates cured with the copolymers after successful purification process (ap).

**Figure 7:**
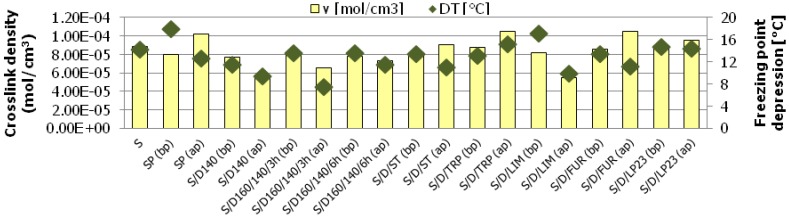
Comparison between crosslinks density of rubber samples calculated from the Flory–Rehner equation based on equilibrium swelling and crosslink density of rubber samples estimated from the freezing point depression results (∆*T*).

The comparison of the DSC thermograms of freezing point depression carried out for rubber samples cured with non-purified and purified sulfur/organic copolymers are given in Figure 6a,b respectively. Figure 7 and Table 3 show the comparison between crosslinks density of rubber samples calculated from the Flory–Rehner equation based on equilibrium swelling data and crosslink density of rubber samples estimated from the freezing point depression results (∆*T*).

The thermograms in Figure 6 show that for pure (free) cyclohexane only one intensive peak of freezing occurs at temperature of approximately 10 °C. Whereas, for all rubber samples swollen in cyclohexane to equilibrium before the analysis and immersed in free cyclohexane in DSC crucible during the analysis, it is visible that two peaks appear. First of them (at higher temperature) corresponds to the freezing of free cyclohexane and the second one (at lower temperature) corresponds to the freezing of cyclohexane that is entrapped in the polymer (rubber) network. In every case both peaks are well separated (not overlapping) and quite developed.

Comparing the crosslink density results with freezing point depression results, it is easy to notice that differences between results obtained from both methods exist. However, in most cases the legitimate trend is retained, which refers to the comparison of the crosslink density calculated from the Flory–Rehner equation and crosslink density changes estimated from the freezing point depression results (∆*T*) between particular pairs of samples. If the ν value is higher for a particular sample (e.g., S/D160/140/6h (bp)) and the value of ∆*T* is also higher and for the sample after purification (S/D160/140/6h (ap)) the both values of ν and ∆*T* are lower, the general trend is legitimate. The almost ideal correspondence was obtained in the case of samples S/D140 (bp) and S/D140 (ap) as well as in the case of samples S/D160/140/6h (bp) and S/D160/140/6h (ap). Reverse (negative) trend was noticed in the case of samples cured with sulfur polymer SP (bp), SP (ap), copolymers based on furfural S/D/FUR (bp), S/D/FUR (ap) and copolymers based on styrene S/D/ST (bp), S/D/ST (ap). The variation of the results from both methods may be attributed to the fact that the network of crosslinked rubber is a complex system, and not only the structure of crosslinks influences the freezing point depression, but also its distribution in the rubber network. Crosslinks in the rubber network are distributed rather randomly and their inhomogeneities may be the result of poor dispersion of curatives during the preparation of rubber mixes [32]. Inhomogeneity of rubber network can also be the result of interactions between crosslinks or presence of chemical groups (i.e., pendant groups from an accelerator) or cyclic structures that are not taking part in the curing process but just modify rubber macromolecules [31,32]. The latter fortunately seems to be negligible in the case of rubber crosslinked with sulfur/organic copolymers after purification (ap). Crioscopic method should not be used independently and the results obtained from freezing point depression ought not to be interpreted directly as crosslink density values.

### 3.5. Mechanical Properties of the Vulcanizates under Static Conditions

**Figure 8:**
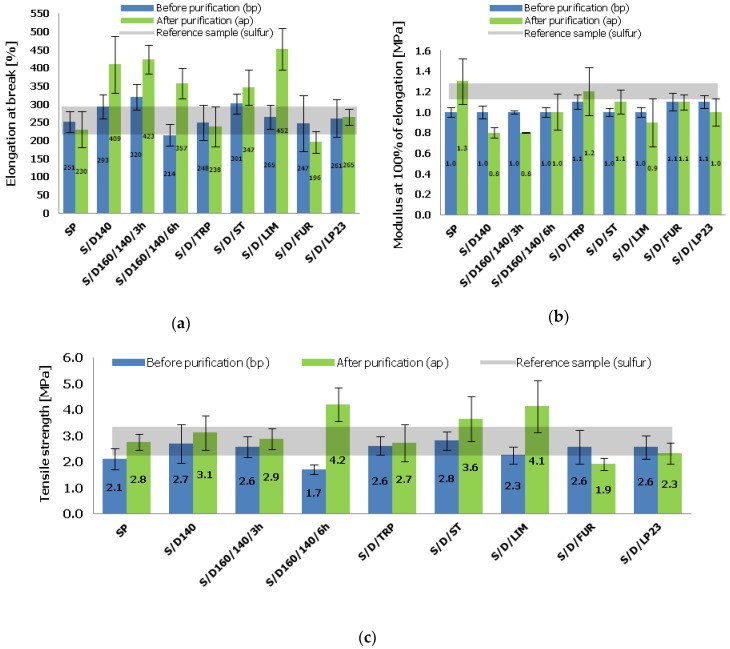
Mechanical properties of the vulcanizates cured with various sulfur/organic copolymers: (**a**) elongation at break (*E*_b_) (%); (**b**) modulus at 100% of elongation (SE100) (MPa); (**c**) tensile strength (*TS*) (MPa).

The tensile tests under static conditions were carried out to study the basic mechanical behavior of the vulcanizates cured with various sulfur/organic copolymers (purified and non-purified) and to compare them also with the reference sample. The results of elongation at break (*E*_b_), modulus at 100% of elongation (SE100) and tensile strength (*TS*) are presented in Figure 8a–c respectively.

The results of elongation at break (*E*_b_) (Figure 8a) suggest that the vulcanizates cured with sulfur/organic copolymers before and after the purification procedure exhibit classical dependence between crosslink density and their elasticity. The samples of lower crosslink density, i.e., S/D140 (ap), S/D160/140/3h (ap) and S/D/LIM (ap) exhibit higher elongation at break (above 400%) and the other way around, the samples of higher crosslink density, i.e., S/D/TRP (ap) and S/D/FUR (ap) exhibit significantly lower elongation at break (below 250%), being also more stiff. Furthermore, in general the vulcanizates cured with purified sulfur/organic copolymers exhibit visibly higher *E*_b_ than before the purification and higher than the reference sample cured with elemental sulfur. Moreover, there is no significant decrease of SE100 (Figure 8b) in the case of vulcanizates cured with purified sulfur/organic copolymers, which means that they are comparably stiff with the reference sample and the samples based on curatives before purification and simultaneously more elastic. Finally, looking at the results of *TS* (Figure 8c), in particular the comparison between the vulcanizates cured with purified and non-purified sulfur/organic copolymers one can see that the purification procedure results in vulcanizates of significantly and surprisingly higher TS, even twice (S/D/LIM) or even over twice higher (S/D160/140/6h). These results may be very attractive, especially from the technical application point of view. The rise of *TS* and improved mechanical properties of the vulcanizates cured with amorphous (purified) sulfur/organic copolymers in comparison to other samples can be attributed to three phenomena: (1) Presumably the curing process takes place without intramolecular modifications of the polymer chains (rubber macromolecules) in the form of cyclic sulfide structures, which are formed during curing with elemental sulfur. In purified sulfur/organic copolymers there is no low molecular sulfur. For this reason (lack of macromolecular modification), a statistically higher amount of curatives is able to take part in the curing process and form chemical bonds between rubber macromolecules. (2) Sulfur/organic copolymers form longer crosslinks than sulfur, which firstly undergo rearrangement and orientation under strain before breakage. This results in higher energy required to break the vulcanizate. (3) Vulcanizates cured with purified sulfur/organic copolymers exhibit more diversified crosslink structures (content of various crosslinks) that allow to achieve better mechanical properties under static conditions [32].

### 3.6. Mechanical Properties of the Vulcanizates under Dynamic Conditions

**Figure 9:**
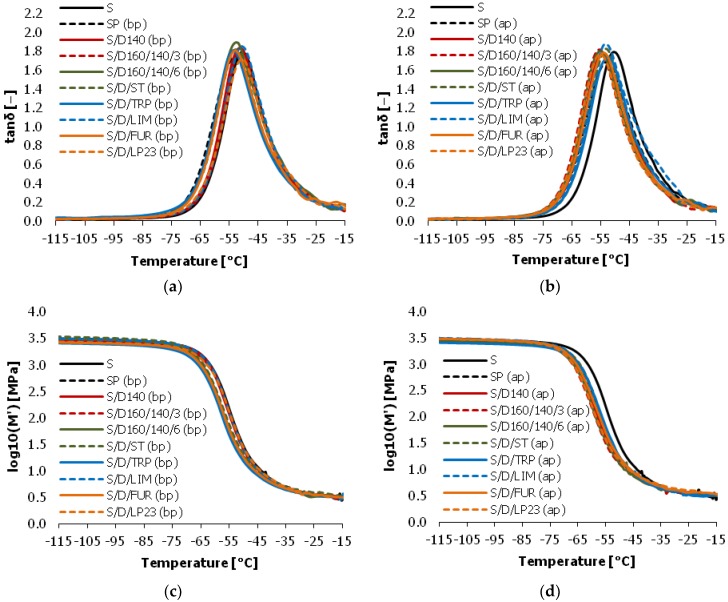

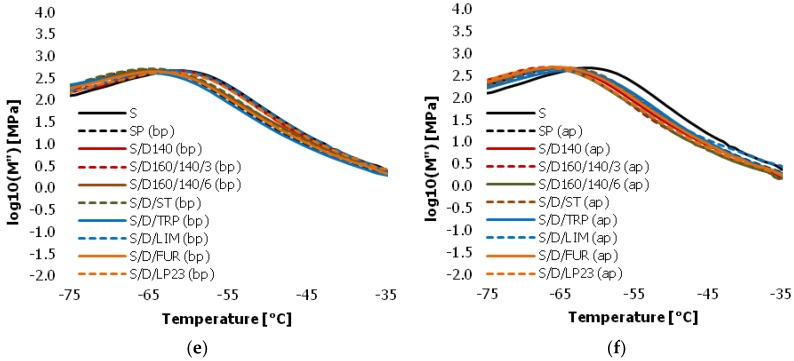
Dynamic mechanical analysis (DMA) analysis of the vulcanizates cured with various sulfur/organic copolymers: (**a**) loss factor (tanδ) of vulcanizates cured with non-purified copolymers; (**b**) loss factor (tanδ) of vulcanizates cured with purified copolymers; (**c**) storage modulus (*M*’) of vulcanizates cured with non-purified copolymers; (**d**) storage modulus (*M*’) of vulcanizates cured with purified copolymers; (**e**) loss modulus (*M*”) of vulcanizates cured with non-purified copolymers; (**f**) loss modulus (*M*”) of vulcanizates cured with purified copolymers.

Dynamic behavior of the cured vulcanizates was tested using dynamic mechanical analysis (DMA). Application of mechanical deformation at particular frequency allows the study of changes in molecular mobility in cured samples as a response to the external forces. The results of DMA parameters values (loss factor (tanδ), storage modulus (*M*’) and loss modulus (*M*”)) in a function of temperature are shown in Figure 9. The full temperature range was truncated in Figure 9 to the temperature range in which the most significant changes of dynamic properties appear. The dynamic glass transition temperature (*T*_g_) was determined as tan delta (tanδ) peak maxima. The values of parameters indicated by DMA analysis are given in Table 4.

Considering the results of glass transition temperature (*T*_g_) we observe that in general the vulcanizates cured with purified sulfur/organic copolymers exhibit lower *T*_g_ than before purification and the reference sample. The *T*_g_ values are lower even up to 5 °C in the case of sample pairs S/D160/140/6h and S/D/LP23. It is most probably the result of different morphology of crosslinks formed during vulcanization with purified copolymers. Sulfur/organic macromolecules form longer and more flexible crosslinks than low molecular sulfur, so they are more mobile and can undergo greater deformation, rearrangement and orientation under external forces. It makes *T*_g_ of the vulcanizates lower. The exception is the sample cured with sulfur/DCPD/turpentine copolymer (S/D/TRP), which *T*_g_ increases when cured with the purified copolymer. This is most probably a result of removing of organic components of turpentine which did not react with sulfur and acted as a plasticizer. Turpentine consists mainly of pinene, which is an unsaturated compound. However, as a bio-solvent obtained from tree resin distillation, it contains also a significant amount of various saturated hydrocarbons which were removed during the copolymer purification in toluene. In general, the values of loss factor (tanδ) of vulcanizates at their glass transition are very similar; however, one can notice that the vulcanizates cured with purified copolymers exhibit slightly lower values of tanδ maxima. This indicates that they may have a little lower ability to suppress vibration.

Interestingly, the rest of the dynamic properties of the vulcanizates (storage modulus (*M*’) and loss modulus (*M*”)) was not affected by the addition of the copolymers as curing agents. This is most probably due to a relatively small amount of the copolymers in the vulcanizates. Storage and loss modulus depend mainly on the SBR rubber matrix properties, which are the same for all the samples. Therefore, various sulfur/organic copolymers may be used for lowering glass transition temperature of the SBR-based vulcanizates without influencing their dynamic characteristics. This introduces the sulfur/organic copolymers as a very interesting alternative for the common sulfur-based curing systems.

## 4. Conclusions

In the present research we used a facile method, the so-called “inverse vulcanization” in order to synthesize chemically stable sulfur-based polymeric materials. The content of polymeric phase in prepared sulfur/organic copolymers as determined by solvent (toluene) extraction is on average 31 wt %, which suggests that spontaneously (thermally) initiated bulk copolymerization of elemental sulfur with unsaturated organic species is not a highly efficient process. However, after solvent extraction (purification) of unreacted sulfur in the form of S_8_ the remained amorphous polymeric phases can be used as curatives for rubber. The comparison of crosslinking with sulfur/organic copolymers before and after the purification procedure taking into account crosslink density values, provided an unambiguous information about participation of sulfur/organic macromolecules in the rubber curing process. Successful crosslinking of SBR rubber with purified sulfur/organic copolymers proved that not only elemental sulfur can be involved in the formation of chemical bonds between rubber macromolecules but also sulfur/organic macromolecules. Moreover, curing with long macromolecules of sulfur/organic copolymers have a significant impact on mechanical properties of the vulcanizates. H. Colvin and Ch. Bull. Jr. in their research [4] claimed that mechanical properties of the vulcanizates depend mainly on the free sulfur content in sulfur/organic copolymers, which according to our results seems to be questionable. Developed procedure of freezing point depression experiments provided valuable information about crosslink density of prepared vulcanizates. In most cases the general trend of crosslink density was consistent with trend determined by equilibrium swelling measurements. However, this method should not be used independently and requires a complementary comparison because it is sensitive not only to crosslink density but also to crosslink structure and macromolecular modifications. Many factors may influence behavior of the solvent trapped in the polymer (rubber) network increasing its complexity. Mechanical properties of the vulcanizates under static conditions reveal that in general vulcanizates cured with sulfur/organic copolymers exhibit classical dependence between crosslink density and elasticity of the materials. However, definitely more interesting mechanical properties are exhibited by vulcanizates cured with purified copolymers due to distinct morphology of their rubber network. Mechanical properties under dynamic conditions show that the vulcanizates cured with purified sulfur/organic copolymers exhibit lower dynamic *T*_g_, which is ascribed to the more flexible and mobile crosslinks. Sulfur/organic copolymers as curatives do not influence the rest of dynamic properties of the vulcanizates in comparison to the reference sample. That makes them very interesting alternative curatives for the common sulfur-based curing systems. In future work we are going to undertake research concerning the influence of sulfur/organic copolymers on sulfur bloom phenomenon.

## Figures and Tables

**Table 1:** Composition of the rubber samples studied.

Ingredient	S and SP (phr)	S/D (phr)	S/D/(e.g., ST) (phr)
SBR KER 1500	100.00	100.00	100.00
Stearic Acid	1.00	1.00	1.00
Zinc Oxide	3.00	3.00	3.00
Sulfur/s-o copolymer	1.52	1.59	1.75
CBS	1.00	1.00	1.00

Abbreviations: S—rubber sample cured with elemental sulfur; SP—rubber sample cured with sulfur polymer; S/D—rubber samples cured with copolymers based on sulfur and dicyclopentadiene; S/D/—rubber samples cured with copolymers based on sulfur, dicyclopentadiene and another comonomer (e.g., styrene, limonene).

**Table 2:** Composition of sulfur/organic copolymers and conditions of (*co*)polymerization process.

Sample Symbol	Organic Comonomer(s)	Sulfur/Organic Comonomer(s) Ratio (wt %)	Reaction Temperature (°C)	Reaction Time (h)	Organoleptic Viscosity
SP	–	100/0	160	3	Low
S/D_140_	DCPD	90.9/9.1	140	3	Low
S/D_160/140/3_	DCPD	90.9/9.1	160/140	1 ^1^/3 ^2^	Low
S/D_160/140/6_	DCPD	90.9/9.1	160/140	1/6	High
S/D/TRP	DCPD/TRP	87/8.7/4.3	140	3	Low
S/D/ST	DCPD/ST	87/8.7/4.3	140	3	High
S/D/LIM	DCPD/LIM	87/8.7/4.3	140	3	Moderate
S/D/FUR	DCPD/FUR	87/8.7/4.3	140	3	Moderate
S/D/LP23	DCPD/LP23	87/8.7/4.3	140	3	Low

^1^ Reaction time after heating sulfur to 160 °C (without comonomers); ^2^ Reaction time after dripping comonomers into liquid sulfur at 140 °C; Abbreviations: DCPD—dicyclopentadiene, TRP—turpentine, ST—styrene, LIM—limonene, FUR—furfural, LP23—liquid rubber Thiokol LP23.

**Table 3:** Values of crosslink density of the rubber samples cured with various sulfur/organic copolymers and their accordance with the freezing point depression results (∆T).

Sample Symbol	ν (mol/cm^3^)	∆*T* (°C)	Accordance of Both Methods
S	8.90 × 10^−5^	14.2	Positive
SP (bp)	8.02 × 10^−5^	18.0	Negative
SP (ap)	1.02 × 10^−4^	12.7	
S/D140 (bp)	7.66 × 10^−5^	11.6	Positive
S/D140 (ap)	5.38 × 10^−5^	9.5	
S/D160/140/3h (bp)	7.96 × 10^−5^	13.7	Positive
S/D160/140/3h (ap)	6.51 × 10^−5^	7.5	
S/D160/140/6h (bp)	7.77 × 10^−5^	13.7	Positive
S/D160/140/6h (ap)	7.33 × 10^−5^	11.6	
S/D/ST (bp)	7.96 × 10^−5^	13.5	Negative
S/D/ST (ap)	9.02 × 10^−5^	11.0	
S/D/TRP (bp)	8.74 × 10^−5^	13.1	Positive
S/D/TRP (ap)	1.05 × 10^−4^	15.2	
S/D/LIM (bp)	8.23 × 10^−5^	17.2	Positive
S/D/LIM (ap)	5.48 × 10^−5^	10.0	
S/D/FUR (bp)	8.60 × 10^−5^	13.5	Negative
S/D/FUR (ap)	1.05 × 10^−4^	11.2	
S/D/LP23 (bp)	8.95 × 10^−5^	14.8	Moderate
S/D/LP23 (ap)	9.49 × 10^−5^	14.5	

**Table 4:** Values of parameters determined by DMA analysis.

Sample Symbol	Dynamic *T*_g_ (°C)	tanδ Maxima (-)
S	−50.6	1.79
SP (bp)	−53.1	1.75
SP (ap)	−53.1	1.68
S/D140 (bp)	−51.3	1.83
S/D140 (ap)	−54.6	1.77
S/D160/140/3h (bp)	−51.0	1.82
S/D160/140/3h (ap)	−55.7	1.81
S/D160/140/6h (bp)	−52.7	1.89
S/D160/140/6h (ap)	−55.0	1.80
S/D/ST (bp)	−53.0	1.81
S/D/ST (ap)	−53.4	1.82
S/D/TRP (bp)	−53.5	1.80
S/D/TRP (ap)	−52.5	1.74
S/D/LIM (bp)	−50.5	1.85
S/D/LIM (ap)	−53.2	1.87
S/D/FUR (bp)	−53.2	1.81
S/D/FUR (ap)	−54.0	1.78
S/D/LP23 (bp)	−50.4	1.71
S/D/LP23 (ap)	−55.4	1.76
